# Perceived Managerial Care, Feedback‐Seeking Behavior, and Reflective Ability in New Nurses: A Three‐Wave Longitudinal Mediation Study

**DOI:** 10.1155/jonm/2288111

**Published:** 2026-07-14

**Authors:** Yongkang Fu, Dongrun Liu, Zhengyi Ma, Hangna Qiu, Juntong Jing, Chaoran Chen, Enshe Jiang, Xiaoguang Zhang

**Affiliations:** ^1^ Department of Neurosurgery, The First Affiliated Hospital of Henan University, Kaifeng, 475004, China, henu.edu.cn; ^2^ Institute of Nursing and Health, School of Nursing and Health, Henan University, Kaifeng, 475004, China, henu.edu.cn

**Keywords:** feedback-seeking behavior, newly graduated nurses, perceived managerial care, reflective ability

## Abstract

**Objective:**

This study aimed to examine the association between perceived managerial care and reflective ability in newly graduated nurses and to investigate the longitudinal mediating role of feedback‐seeking behavior, providing a theoretical basis for managerial interventions.

**Background:**

Reflective ability is a critical competence for newly graduated nurses to adapt to complex clinical environments and promote professional development. Managerial care has been shown to influence nurses’ learning‐related behaviors; however, the longitudinal mechanisms through which perceived managerial care enhances reflective ability remain unclear.

**Design:**

A three‐wave longitudinal survey design was employed, with data collected at three time points (T1, T2, and T3) over 6 months.

**Methods:**

Newly graduated nurses from 10 tertiary general hospitals in China were recruited using simple random sampling. Validated scales were used to assess perceived managerial care, feedback‐seeking behavior, and reflective ability. Structural equation modeling and bootstrapping analyses were conducted to examine longitudinal mediation effects.

**Results:**

Perceived managerial care, feedback‐seeking behavior, and reflective ability were positively correlated at T1, T2, and T3 (*p* < 0.01), with mean scores increasing over time. Feedback‐seeking behavior played a significant longitudinal mediating role between perceived managerial care and reflective ability, accounting for 44.02% of the total effect. The structural equation model showed acceptable fit indices.

**Conclusion:**

Feedback‐seeking behavior mediates the relationship between perceived managerial care and reflective ability in newly graduated nurses. Strengthening managerial care and encouraging feedback‐seeking behaviors may effectively promote reflective ability and support early career development.

**Implications for Nursing Management:**

The findings suggest that nursing managers may enhance new nurses’ reflective ability by fostering supportive managerial environments and encouraging feedback‐seeking behavior. Strengthening managerial care and establishing effective feedback mechanisms could facilitate professional development and improve early career adaptation among new nurses.

## 1. Introduction

In the context of rapid advances in medicine and increasingly diverse patient needs, nursing practice is continuously evolving toward greater precision, personalization, and interdisciplinary collaboration. As a result, the complexity of nursing work has increased substantially, placing more stringent demands on nurses’ comprehensive competencies [1, 2]. Newly graduated nurses—defined as nurses who have just entered clinical practice and have less than 2 years of work experience—represent a vital emerging force within the nursing workforce and are at a critical transitional stage from academic learning to professional practice [3]. In addition to possessing solid professional knowledge and technical skills, they must continually update their ways of thinking to cope with rapidly changing clinical situations [4, 5]. Therefore, research focusing on newly graduated nurses is of great significance. Such research not only facilitates a deeper understanding of the problems and challenges they encounter during the early stage of employment, thereby providing a solid foundation for targeted support and training, but also offers valuable insights for optimizing and improving nursing talent development systems [6].

Reflective ability is a core competency that enables nurses to enhance self‐awareness, optimize practice, and adapt to complex healthcare environments [7]. It refers to an individual’s capacity to critically think about, analyze, and evaluate their own behaviors, decisions, and outcomes during practice [8]. In the field of nursing, reflective ability is particularly important, as it helps nurses identify problems and deficiencies in practice, facilitates the deepening of knowledge and improvement of skills, and ultimately promotes professional growth and better fulfillment of patients’ health needs [9, 10]. Newly graduated nurses are at a crucial stage of learning and development, and the cultivation and enhancement of their reflective ability are essential for their professional growth. Examining reflective ability among newly graduated nurses can help identify its influencing factors and enable the development of targeted strategies to promote its improvement, thereby facilitating their transition into competent nursing professionals with strong professional and ethical qualities [11]. Accordingly, understanding the current status, influencing factors, and developmental pathways of nurses’ reflective ability is of profound significance for optimizing nursing management, improving the quality of nursing services, and promoting nurses’ career development.

In recent years, research on reflective ability has increased across various fields and professional groups [12, 13]. Within the nursing domain, scholars have conducted preliminary explorations of nurses’ reflective ability. Existing studies suggest that reflective ability is closely related to factors such as work experience, educational background, and the work environment [14–16]. However, several limitations remain. On the one hand, studies focusing specifically on reflective ability among newly graduated nurses are relatively scarce. More importantly, a systematic theoretical framework explaining the factors influencing their reflective ability has yet to be established. Most existing studies examine isolated factors and lack model‐based analyses that capture the intrinsic relationships among variables [17]. On the other hand, prior research has predominantly adopted cross‐sectional designs, which can only reveal associations between reflective ability and outcome variables such as professional cognition and nursing quality, without further elucidating deeper mechanisms, including how specific factors influence reflective ability and which variables play mediating or moderating roles within a theoretical framework [18, 19]. Therefore, the present study constructs a mediation model and adopts a longitudinal design to explore the mechanisms through which perceived managerial care and feedback‐seeking behavior influence reflective ability among newly graduated nurses, to provide new perspectives and theoretical support for enhancing nurses’ reflective ability.

## 2. Background

In today’s complex and dynamic nursing work environment, nurses are confronted with high levels of work pressure and professional challenges. Perceived managerial care, as an important concept in nursing practice, has received increasing scholarly attention in recent years. Perceived managerial care refers to nurses’ most direct perceptions of the external organizational environment, specifically their perceived level of concern, support, and understanding provided by nursing managers [20]. This perception encompasses not only emotional care but also support for career development and improvements in the work environment [21]. Research has shown that perceived managerial care can significantly enhance nurses’ job satisfaction, alleviate burnout, and increase work engagement [22]. Social cognitive theory posits that individual behavior is shaped by the interaction between environmental and personal factors [23, 24]. A high level of perceived managerial care can provide nurses with emotional support and professional guidance [25], help alleviate work‐related stress, and strengthen self‐confidence, which may contribute to the development of reflective ability [26]. Moreover, perceived managerial care may further enhance reflective ability by optimizing the work environment and providing nurses with more opportunities for learning and growth [27].

Feedback‐seeking behavior refers to individuals’ proactive efforts to obtain information from others regarding their behaviors, performance, or work outcomes [28]. In the nursing context, feedback‐seeking behavior among newly graduated nurses manifests as actively seeking input from colleagues, head nurses, patients, and other stakeholders regarding the accuracy of nursing procedures, the appropriateness of care plans, and the strengths and weaknesses of their professional performance [29, 30]. Feedback‐seeking behavior is an important pathway through which nurses adapt to the work environment and enhance their professional skills and competencies [31]. Previous studies indicate that perceived managerial care can enhance employees’ sense of psychological safety [32]. As newly graduated nurses are relatively unfamiliar with workplace environments and job requirements, perceiving managerial care may foster a sense of organizational belonging, thereby encouraging them to express their uncertainties and needs more openly [33], which in turn may facilitate feedback‐seeking behavior [34]. In addition, perceived managerial care can stimulate nurses’ motivation for self‐improvement [35]. When newly graduated nurses perceive that management is concerned about their professional development, they may be more inclined to seek feedback to enhance their competencies and meet organizational expectations [36]. In other words, when newly graduated nurses perceive managerial care, they may be more willing to proactively seek feedback.

As one of the core elements of nurses’ professional development, the enhancement of reflective ability does not occur instantaneously; rather, it requires newly graduated nurses to engage in proactive exploration, active learning, and continuous self‐examination [37]. Feedback‐seeking behavior constitutes a critical component of this process. By actively seeking feedback, nurses can obtain multidimensional information about their practice, which provides rich material and diverse perspectives for reflection [38]. For example, feedback from head nurses can help newly graduated nurses understand the standardization of nursing procedures, whereas feedback from patients and their families can reveal deficiencies in the humanistic aspects of nursing care [39]. Such feedback offers concrete issues and directions for improvement, thereby potentially enhancing reflective ability. Some studies have also shown that newly graduated nurses often lack accurate self‐assessment when they first enter clinical practice [40]. By actively seeking feedback, they may obtain more objective information from others’ evaluations, enabling them to adjust their self‐perceptions accordingly [41]. This more objective self‐evaluation may render their reflection more rational and, in turn, promote the development of reflective ability.

**FIGURE 1:**
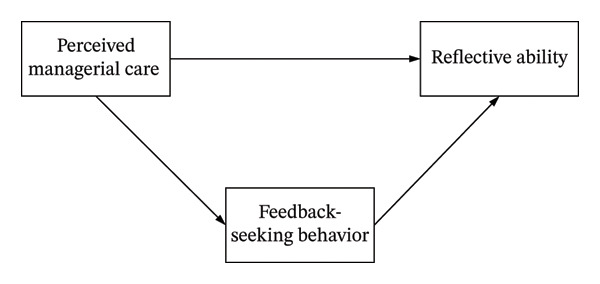
Theoretical framework.

In summary, we propose that perceived managerial care can promote the enhancement of reflective ability among newly graduated nurses, with feedback‐seeking behavior playing a critical mediating role in this relationship. However, systematic empirical examinations of this mechanism remain limited. Particularly within a longitudinal framework, exploring how perceived managerial care influences reflective ability through feedback‐seeking behavior is of substantial importance for both theoretical advancement and practical application. Therefore, we propose the theoretical framework of the present study (Figure 1) and construct a longitudinal mediation model involving perceived managerial care, feedback‐seeking behavior, and reflective ability among newly graduated nurses to examine the mechanisms underlying these relationships. Based on this framework, the following hypotheses are proposed:

 Hypothesis 1: Perceived managerial care positively predicts reflective ability among newly graduated nurses in the model.
 Hypothesis 2: Perceived managerial care positively predicts feedback‐seeking behavior among newly graduated nurses in the model.
 Hypothesis 3: Feedback‐seeking behavior positively predicts reflective ability among newly graduated nurses in the model.
 Hypothesis 4: Feedback‐seeking behavior plays a longitudinal mediating role in the relationship between perceived managerial care and reflective ability among newly graduated nurses.

## 3. Methods

### 3.1. Participants and Procedure

#### 3.1.1. Research Design

This study adopted a longitudinal questionnaire‐based design. By collecting data from newly graduated nurses at multiple time points, the study aimed to systematically examine the dynamic relationships among perceived managerial care, feedback‐seeking behavior, and reflective ability, thereby providing robust empirical evidence for exploring potential prospective associations among these variables. Data were collected using a self‐reported method across three time points (T1, T2, and T3). In addition, a structural equation model (SEM) was constructed to test the mediating role of feedback‐seeking behavior. This study was conducted and reported in accordance with the STROBE guidelines to ensure transparency and reporting quality.

### 3.2. Participants

Participants were recruited from 10 general hospitals in China with varying levels of economic development and healthcare resource allocation. Within the selected hospitals, a simple random sampling method was used to recruit newly graduated nurses for the questionnaire survey. The inclusion criteria were as follows: (1) nurses who had obtained professional qualification certificates and completed registration; (2) voluntary participation with informed consent; and (3) work experience of no more than 1.5 years. The exclusion criteria were as follows: (1) nurses with prior work experience before their current position; (2) questionnaires with substantial missing data, omissions, or invalid responses; and (3) nurses who were on long‐term leave, such as maternity leave or sick leave. Before data collection, permission was obtained from hospital administrators, and the questionnaires were distributed to the hospitals. In addition to the questionnaires, detailed instructions on how to complete them were provided to ensure clarity and accuracy of responses and to ensure the ethical conduct of the research.

### 3.3. Sample Size

A priori power analysis was conducted using a Monte Carlo simulation based on the method proposed by Schoemann et al. [42] to determine the minimum required sample size. In the Monte Carlo simulation, the target statistical power was set at 0.80, with 1000 replications to ensure the stability of the results. Each replication involved 5000 Monte Carlo draws to generate a reliable sampling distribution of indirect effects. A random seed of 1234 was specified to ensure reproducibility, and the confidence interval (CI) width was set at 95% (corresponding to *α* = 0.05). Based on the correlations and standard deviations of the variables in the model, the minimum required sample size was estimated to be 374. Considering a potential attrition rate of 20%, as well as the need to ensure the precision of parameter estimation, account for potentially invalid questionnaires during data processing, and provide sufficient statistical power for subsequent model analyses, a total of 500 newly graduated nurses were ultimately recruited for this study.

### 3.4. Data Collection

A longitudinal follow‐up design was employed, with data collected at three time points: June 2025 (T1), September 2025 (T2), and December 2025 (T3). The 3‐month interval between the three measurement waves was determined based on the following considerations. On the one hand, this interval is broadly consistent with routine clinical rotation and training cycles in hospital settings, which facilitates participant follow‐up and enhances the feasibility, stability, and consistency of longitudinal data collection. On the other hand, changes in feedback‐seeking behavior and reflective ability require a certain period of accumulation. If the interval is too short, it may be difficult to adequately capture cross‐time changes and lagged effects among the variables; if the interval is too long, additional potential confounding factors may be introduced, thereby weakening the temporal associations among variables. Using simple random sampling, 50 newly graduated nurses were selected from each of the 10 participating tertiary general hospitals, resulting in a total sample of 500 participants. The survey instruments included a demographic questionnaire, the perceived managerial care scale, the feedback‐seeking behavior scale, and the reflective ability scale. To ensure the traceability and accurate matching of longitudinal data, each participant was assigned a unique anonymous identification number (ID) at T1, which was consistently used at T2 and T3 to ensure accurate correspondence of data across time points. At T1, 500 questionnaires were distributed, and 467 valid questionnaires were returned. Three months after the completion of the T1 survey, the second wave of data collection (T2) was conducted. After excluding cases with missing data, mismatched IDs across time points, and patterned responses, 436 valid questionnaires were obtained. Three months after the T2 survey, the third wave of data collection (T3) was conducted using the same procedures and exclusion criteria as in T2, yielding 414 valid questionnaires in the final sample (effective response rate = 82.80%). During data processing, cross‐time ID matching checks were performed to ensure that data from the same participants were accurately and completely matched across T1, T2, and T3. These systematic quality control measures ensured the reliability and rigor of the study data.

### 3.5. Measures

#### 3.5.1. Caring Assessment Tool–Administration

Perceived managerial care was measured using the Caring Assessment Tool–Administration developed by Wolverton et al. [20] and translated and revised into Chinese by Peng et al. [43]. The scale consists of 36 items across three dimensions: shared decision‐making (12 items), respect (14 items), and lack of caring (10 items). Responses are rated on a five‐point Likert scale ranging from 1 (“never”) to 5 (“always”), with the lack‐of‐caring dimension reverse scored. Higher total scores indicate higher levels of perceived managerial care among newly graduated nurses. In the present study, Cronbach’s α coefficients for this scale at T1, T2, and T3 were 0.987, 0.979, and 0.986, respectively, indicating excellent internal consistency.

#### 3.5.2. Feedback Seeking Questionnaire

Feedback‐seeking behavior was assessed using the Feedback Seeking Questionnaire developed by Callister [44] and translated and revised by Gong [45]. The scale includes 11 items encompassing four dimensions: leader monitoring feedback seeking (2 items; Items 1–2), leader inquiry feedback seeking (2 items; Items 3–4), coworker inquiry feedback seeking (4 items; Items 5–8), and coworker monitoring feedback seeking (3 items; Items 9–11). Items are rated on a seven‐point Likert scale ranging from 1 (“strongly disagree”) to 7 (“strongly agree”). Total scores range from 11 to 77, with higher scores indicating more frequent feedback‐seeking behavior. In this study, Cronbach’s α coefficients at T1, T2, and T3 were 0.946, 0.948, and 0.950, respectively, indicating high internal consistency.

#### 3.5.3. Reflective Ability Scale for Clinical Nurses

Reflective ability was measured using the Reflective Ability Scale for Clinical Nurses developed by Nishimoto et al. [9] and translated and revised by Shao et al. [46]. The scale comprises 19 items across three dimensions: reviewing nursing practice, reflecting on nursing practice, and extending nursing practice. Responses are rated on a five‐point Likert scale ranging from 1 (“strongly disagree”) to 5 (“strongly agree”), yielding a total score range of 19–95. Higher scores indicate stronger reflective ability among clinical nurses. In the present study, Cronbach’s α coefficients at T1, T2, and T3 were 0.883, 0.871, and 0.879, respectively, indicating good internal consistency.

### 3.6. Data Analysis

Data were analyzed using IBM SPSS 25.0 and AMOS 26.0. First, Harman’s single‐factor test was conducted to assess common method bias (CMB), and frequencies and percentages were used to describe participants’ demographic characteristics. Second, Pearson’s correlation analysis was performed to examine the relationships among perceived managerial care, feedback‐seeking behavior, and reflective ability, and descriptive statistics were calculated for these variables at T1, T2, and T3. When variables did not meet the assumption of normality, Spearman’s correlation analysis was applied. Finally, structural equation modeling was conducted using AMOS 26.0. In the model, perceived managerial care served as the independent variable, reflective ability as the dependent variable, and feedback‐seeking behavior as the mediating variable. Model fit was evaluated using the chi‐square (*χ* ^2^) statistic and several fit indices, including the GFI, CFI, and RMSEA. Acceptable model fit was indicated by CFI and TLI values of 0.90 or higher and RMSEA values of 0.08 or lower [47]. Bootstrapping with 5000 resamples was used to estimate multiple mediation effects and bias‐corrected CIs. A mediating effect was considered significant if the 95% CI did not include zero. Two‐tailed *p* values less than 0.05 were regarded as statistically significant.

### 3.7. Ethical Considerations

This study was conducted as an anonymous survey and did not involve unethical practices or human clinical experiments, nor did it pose any physical or psychological harm to participants. The study was carried out in accordance with the Declaration of Helsinki and was reviewed and approved by the Ethics Subcommittee for Biological and Scientific Research of Henan University (Approval No. HUSOM2025‐985). Before questionnaire distribution, informed consent was obtained from the participating hospitals and nursing departments. Participation was entirely voluntary, and all data were treated with strict confidentiality.

## 4. Results

### 4.1. CMB Test

As all variables in this study were obtained through self‐reported measures, it was necessary to examine the potential presence of CMB. Harman’s single‐factor test was conducted, and the results showed that, across the three measurement waves (T1, T2, and T3), the first factor accounted for 39.94%, 37.84%, and 40.98% of the total variance, respectively, all of which were below the threshold of 50%. Therefore, no serious CMB was detected in this study [48].

### 4.2. The Demographic Characteristics of the Participants

A total of 414 nurses participated in this study, including 115 males (27.8%) and 299 females (72.2%). Among the participants, 72.4% held a bachelor’s degree or higher, and the majority of newly graduated nurses were contract employees (60.6%). More than half of the participants reported a monthly income of less than 8000 RMB (63.0%).

In addition, to assess whether sample attrition introduced systematic bias into the study findings, independent‐sample *t*‐tests and chi‐square tests were conducted to compare demographic characteristics and key study variables, including perceived managerial care, feedback‐seeking behavior, and reflective ability, between participants who completed all three waves of data collection and those who dropped out during the study. The results indicated no significant differences between the retained and attrition groups on any of these variables (all *p* > 0.05), suggesting that sample attrition did not introduce substantial systematic bias. Therefore, the final sample can be considered adequately representative, supporting the appropriateness of subsequent longitudinal analyses. Detailed demographic characteristics are presented in Table 1.

**TABLE 1:** Participants’ background and comparison between those who completed and those who withdrew from the study.

Demographics	Full participated (*n* = 414) *N* (%)/mean ± S.D.	Withdrew (*n* = 53) *N* (%)/mean ± S.D.	*p*
Gender			0.618
Male	115 (27.8%)	13 (24.5%)	
Female	299 (72.2%)	40 (75.5%)	
Education			0.163
≤ Associate’s degree	73 (17.6)	10 (18.9%)	
Bachelor’s degree	273 (65.9)	29 (54.7%)	
≥ Master’s degree	68 (16.5)	14 (26.4%)	
Department			0.380
Internal medicine department	111 (26.8)	8 (15.1)	
Surgery department	81 (19.6)	13 (24.5)	
Gynecology and obstetrics	34 (8.2)	4 (7.5)	
Pediatric department	22 (5.3)	3 (5.8)	
Emergency department	35 (8.4)	4 (7.5)	
Intensive care unit	26 (6.3)	7 (13.2)	
Others	105 (25.4)	14 (26.4)	
Labor and personnel relations			0.176
Regular establishment staff	83 (20.1)	10 (18.9)	
Personnel agency	80 (19.3)	16 (30.2)	
Contract worker	251 (60.6)	27 (50.9)	
Average monthly income (RMB)			0.454
≤ 8000	261 (63.0)	39 (73.6)	
8000∼10,000	124 (30.0)	11 (20.8)	
1000∼15,000	14 (3.4)	2 (3.8)	
> 15,000	15 (3.6)	1 (1.8)	
Perceived managerial care	120.77 ± 37.70	121 ± 39.25	0.379
Feedback‐seeking behavior	45.75 ± 12.86	44.92 ± 11.74	0.180
Reflective ability	64.72 ± 13.64	67.26 ± 12.78	0.375

### 4.3. Correlation Analysis and Significance Testing

Pearson’s correlation coefficients for all variables are presented in Table 2. Perceived managerial care, feedback‐seeking behavior, and reflective ability among newly graduated nurses were all significantly and positively correlated with one another at T1, T2, and T3 (*p* < 0.01). These findings indicate that the concurrent and lagged correlations among perceived managerial care, feedback‐seeking behavior, and reflective ability were generally consistent, satisfying the prerequisites for constructing a longitudinal mediation SEM and conducting cross‐lagged analyses in the present study.

**TABLE 2:** Correlations among the study variables (*N* = 414).

	T1PMC	T2PMC	T3PMC	T1FSB	T2FSB	T3FSB	T1RA	T2RA	T3RA
T1PMC	1								
T2PMC	0.370^∗∗^	1							
T3PMC	0.269^∗∗^	0.511^∗∗^	1						
T1FSB	0.267^∗∗^	0.381^∗∗^	0.323^∗∗^	1					
T2FSB	0.303^∗∗^	0.483^∗∗^	0.447^∗∗^	0.581^∗∗^	1				
T3FSB	0.157^∗∗^	0.349^∗∗^	0.311^∗∗^	0.326^∗∗^	0.442^∗∗^	1			
T1RA	0.294^∗∗^	0.494^∗∗^	0.428^∗∗^	0.620^∗∗^	0.585^∗∗^	0.263^∗∗^	1		
T2RA	0.183^∗∗^	0.218^∗∗^	0.198^∗∗^	0.408^∗∗^	0.400^∗∗^	0.494^∗∗^	0.367^∗∗^	1	
T3RA	0.342^∗∗^	0.369^∗∗^	0.431^∗∗^	0.461^∗∗^	0.555^∗∗^	0.359^∗∗^	0.487^∗∗^	0.382^∗∗^	1

Abbreviations: FSB, feedback‐seeking behavior; PMC, perceived managerial care; RA, reflective ability.

^∗∗^ *p* < 0.01 (two‐tailed).

### 4.4. Descriptive Statistics of Study Variables

Descriptive statistics for all study variables at T1, T2, and T3 are presented in Table 3. The mean scores for perceived managerial care were 120.77 ± 37.70 at T1, 125.41 ± 33.24 at T2, and 127.25 ± 36.70 at T3. The corresponding scores for feedback‐seeking behavior were 45.75 ± 12.86, 47.65 ± 13.27, and 48.02 ± 14.22, respectively, while the scores for reflective ability were 64.72 ± 13.64, 65.46 ± 12.60, and 66.33 ± 13.22, respectively. Compared with T1, perceived managerial care, feedback‐seeking behavior, and reflective ability all showed increases at T2, and further increases were observed at T3 relative to T2. In addition, all autoregressive paths among the study variables were statistically significant (*p* < 0.001), and Cronbach’s α coefficients for each variable at T1, T2, and T3 were all greater than 0.80, confirming the stability and reliability of the measures used in this study.

**TABLE 3:** Scores for research variables (*N* = 414).

Variables		Data collection			Autoregressive path	
		T1	T2	T3	T1 ⟶ T2	T2 ⟶ T3
PMC	M	120.77	125.41	127.25	*β* = 0.37	*β* = 0.51
	SD	37.70	33.24	36.70	*p* < 0.001	*p* < 0.001
	*α*	0.987	0.979	0.986		

FSB	M	45.75	47.65	48.02	*β* = 0.53	*β* = 0.37
	SD	12.86	13.27	14.22	*p* < 0.001	*p* < 0.001
	*α*	0.946	0.948	0.950		

RA	M	64.72	65.46	66.33	*β* = 0.21	*β* = 0.17
	SD	13.64	12.60	13.22	*p* < 0.001	*p* = 0.001
	*α*	0.883	0.871	0.879		

*Note:* α: Cronbach’s α.

Abbreviations: FSB, feedback‐seeking behavior; M, mean; PMC, perceived managerial care; RA, reflective ability; SD, standard deviation.

### 4.5. Mediation Analysis

Using AMOS 26.0, a SEM was constructed with perceived managerial care as the independent variable, feedback‐seeking behavior as the mediating variable, and reflective ability as the dependent variable to examine the mediating role of feedback‐seeking behavior in the relationship between perceived managerial care and nurses’ reflective ability. The results indicated that the model demonstrated a good fit to the data (CMIN/DF = 3.476, RMSEA = 0.077, GFI = 0.982, CFI = 0.981, TLI = 0.930, IFI = 0.981). Detailed results are presented in Table 4.

**TABLE 4:** Structural equation model fitting index.

Model	CMIN/DF	RMSEA	GFI	CFI	TLI	IFI
Recommended value	< 5	< 0.08	> 0.90	> 0.90	> 0.90	> 0.90
Mediation model	3.476	0.077	0.982	0.981	0.930	0.981

*Note:* CMIN/DF, Chi‐square divided by degrees of freedom.

Abbreviations: CFI, comparative fit index; GFI, goodness‐of‐fit index; IFI, incremental fit index; RMSEA, root mean square error of approximation; TLI, Tucker–Lewis index.

**FIGURE 2:**
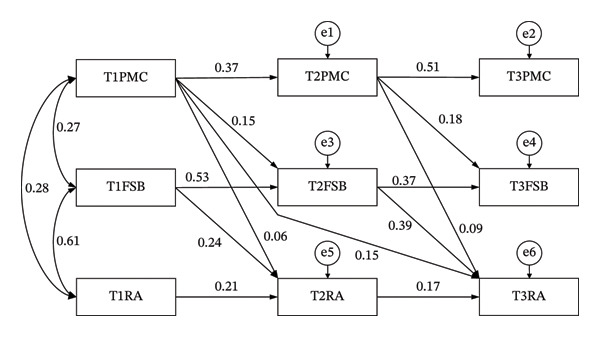
A longitudinal mediating model of perceived managerial care, feedback‐seeking behavior, and reflective ability. PMC: perceived managerial care; FSB: feedback‐seeking behavior; RA: reflective ability.

Path analysis results are illustrated in Figure 2. Perceived managerial care at T1 (T1 PMC) significantly and positively predicted feedback‐seeking behavior at T2 (T2 FSB; *β* = 0.15, *p* < 0.001) and reflective ability at T2 (T2 RA; *β* = 0.06, *p* < 0.001). Feedback‐seeking behavior at T1 (T1 FSB) significantly and positively predicted reflective ability at T2 (*β* = 0.24, *p* < 0.001). Furthermore, perceived managerial care at T2 (T2 PMC) significantly and positively predicted feedback‐seeking behavior at T3 (T3 FSB; *β* = 0.18, *p* < 0.001) and reflective ability at T3 (T3 RA; *β* = 0.09, *p* < 0.001). Feedback‐seeking behavior at T2 (T2 FSB) also significantly and positively predicted reflective ability at T3 (*β* = 0.39, *p* < 0.001), thereby supporting Hypotheses 1, 2, and 3. In addition, perceived managerial care at T1 directly and positively predicted reflective ability at T3 (*β* = 0.15, *p* < 0.001). The indirect pathway from perceived managerial care to reflective ability through feedback‐seeking behavior was significant, confirming Hypothesis 4.

Finally, a bootstrapping procedure with 5000 resamples was employed to examine the mediating role of feedback‐seeking behavior in the relationship between perceived managerial care and reflective ability among newly graduated nurses. The results indicated that feedback‐seeking behavior played a significant mediating role between perceived managerial care and reflective ability, as the 95% CI for the total indirect effect did not include zero. The indirect effect was 0.151 (*p* < 0.001; bootstrap 95% CI: 0.092, 0.216), accounting for 44.02% of the total effect. The direct effect of perceived managerial care on reflective ability was 0.192 (*p* < 0.001; bootstrap 95% CI: 0.109, 0.274), accounting for 55.98% of the total effect. Detailed results are presented in Table 5.

**TABLE 5:** Bootstrap analysis of the mediating model.

Effects	Paths	Effect	SE	95% CI	*p*	Percentage
Total effect	T1PMC ⟶ T3RA	0.343	0.046	0.252 to 0.434	< 0.001	100%
Direct effect	T1PMC ⟶ T3RA	0.192	0.042	0.109 to 0.274	< 0.001	55.98%
Indirect effect	T1PMC ⟶ T2FSB ⟶ T3RA	0.151	0.032	0.092 to 0.216	< 0.001	44.02%

Abbreviations: FSB, feedback‐seeking behavior; PMC, perceived managerial care; RA, reflective ability.

## 5. Discussion

Using a three‐wave longitudinal design, this study constructed a longitudinal mediation model to examine the effect of perceived managerial care on new nurses’ reflective ability and the mediating role of feedback‐seeking behavior. All proposed hypotheses were supported. The results of the three‐wave follow‐up survey showed that the autoregressive coefficients of perceived managerial care were 0.37 from T1 to T2 and 0.51 from T2 to T3, indicating a certain degree of temporal stability that gradually strengthened over time. This finding may reflect the sustained nature of perceived managerial care over a short‐term time frame. The autoregressive coefficients of feedback‐seeking behavior were 0.53 from T1 to T2 and 0.37 from T2 to T3. These results indicate that feedback‐seeking behavior demonstrates relatively high stability during the early career stage of new nurses and is positively associated with reflective ability. However, this stability appears to weaken at later stages, suggesting that feedback‐seeking behavior may be increasingly affected by other factors, thereby weakening its positive relationship with reflective ability [49, 50]. The autoregressive coefficients of reflective ability were 0.21 from T1 to T2 and 0.17 from T2 to T3, indicating relatively low and gradually decreasing stability. This finding implies that reflective ability changes more rapidly over time and shows weaker associations with its prior levels, relying more heavily on external factors, such as perceived managerial care and feedback‐seeking behavior [51]. Additionally, this finding suggests that the development of reflective ability is context‐dependent; rather than emerging spontaneously, it relies on sustained organizational support and active behavioral engagement. Based on these findings, it is recommended that nursing managers establish positive moral role models during the early onboarding stage of new nurses. By providing strong perceived managerial care and support, managers can enhance new nurses’ feedback‐seeking behavior, thereby promoting the development of reflective ability. Moreover, continuous attention should be paid to strengthening feedback‐seeking behavior throughout new nurses’ career development to sustain improvements in reflective ability and support their professional growth.

The results also demonstrated that perceived managerial care was significantly and positively associated with new nurses’ reflective ability. Greater perceived managerial care was associated with higher levels of reflective ability, which is consistent with previous research. For example, Tian et al. found that employees with higher levels of perceived managerial care exhibited stronger learning motivation and self‐improvement intentions, thereby facilitating the development of reflective ability [52]. Several mechanisms may explain this relationship. First, perceived managerial care enhances psychological safety. As new nurses enter the workforce, they often experience considerable psychological pressure due to complex work environments and high job demands [2, 53]. When they perceive care and support from management, they are more confident in expressing their ideas and concerns [54], which in turn encourages reflective engagement [55]. Second, perceived managerial care stimulates self‐improvement motivation [56]. Managerial attention and expectations motivate new nurses to critically examine their work performance, identify shortcomings through reflection, and implement improvements, thereby enhancing reflective ability [37]. Finally, it should be noted that this relationship may also be influenced by other potential factors. For example, individual characteristics (such as differences in economic conditions and lifestyle indicators) may simultaneously affect perceived managerial care and reflective ability. In addition, factors such as team climate or organizational climate may also play a role. Therefore, future research should incorporate multilevel variables to enhance the explanatory power of the model. Accordingly, hospital administrators are advised to promote a culture of care from a strategic perspective by establishing onboarding care programs for new nurses, including systematic pre‐employment training, mentorship arrangements, and regular psychological counseling. As direct supervisors, nurse managers should maintain frequent communication to understand new nurses’ work and life conditions, provide emotional support, and organize team‐sharing sessions to encourage the exchange of work experiences and reflective outcomes. These efforts can foster a supportive managerial environment and promote the development of reflective ability among new nurses.

Finally, the findings indicate that feedback‐seeking behavior mediates the relationship between perceived managerial care and new nurses’ reflective ability. Specifically, new nurses who perceive higher levels of managerial care are more inclined to engage in feedback‐seeking behavior, which provides rich material for reflection and, in turn, promotes reflective ability. This finding is consistent with prior research suggesting that perceived managerial care can enhance employees’ learning behaviors and capability development by increasing work engagement [57]. Extending previous research, the present study further elucidates the mediating role of feedback‐seeking behavior in this process, particularly among new nurses in the nursing profession. Perceived managerial care may enhance new nurses’ feedback‐seeking behavior by fostering psychological safety and a sense of belonging [58]. Under such conditions, new nurses are less fearful of exposing their weaknesses [59] and are more likely to view feedback as an opportunity for growth, motivating them to proactively seek feedback to improve their work performance [50]. Supported by psychological safety and belongingness, new nurses are more inclined to seek feedback from colleagues, supervisors, patients, or patients’ families [60]. Through analyzing feedback content, new nurses can gain deeper insights into the relationships between their behaviors and nursing outcomes, thereby enhancing reflective ability [61]. Kolb’s experiential learning theory further posits that learning is a cyclical process consisting of concrete experience, reflective observation, abstract conceptualization, and active experimentation [62]. Feedback‐seeking behavior provides new nurses with abundant external information [63], which serves as critical input during the reflective observation stage, facilitating a deeper understanding of the links between actions and outcomes and promoting the development of reflective ability [63–65]. Based on these findings, hospital administrators are encouraged to establish formal feedback mechanisms, encourage proactive feedback‐seeking among new nurses, and respond promptly to their needs. Additionally, regular nursing workshops can be organized to invite experts to share clinical experiences and feedback strategies, helping new nurses make more effective use of feedback for reflection. Nurse managers may also provide clear feedback channels, such as feedback boxes or regular team feedback meetings, to encourage experience sharing and reflective dialogue. Finally, new nurses themselves may form peer support groups to regularly exchange experiences and feedback, thereby enhancing reflective ability through collective learning.

## 6. Significance to Practice

This study elucidates the intrinsic relationships among perceived managerial care, feedback‐seeking behavior, and new nurses’ reflective ability, providing novel theoretical insights and practical implications for nursing management, education, and training. First, the findings demonstrate that perceived managerial care can positively stimulate feedback‐seeking behavior, thereby facilitating the systematic development of reflective ability. This offers actionable strategies for refined nursing management. By establishing open feedback mechanisms and strengthening emotional and professional support for new nurses, nursing managers can encourage proactive feedback‐seeking behavior. Such practices not only enhance team collaboration and foster a learning‐oriented climate but also strengthen new nurses’ professional identity and sense of belonging, reduce burnout and turnover risk, and ultimately improve overall nursing quality and efficiency.

Second, this study provides new directions for optimizing nursing education and training. Feedback‐seeking behavior and reflective ability are core competencies in nursing practice and key objectives of nursing education. The demonstrated positive role of perceived managerial care suggests that nursing educators may integrate managerial care concepts into standardized training programs, such as through simulated management scenarios or caring‐oriented mentorship, helping new nurses develop proactive feedback‐seeking awareness and reflective habits early in their careers. Finally, the empirical findings offer targeted decision‐making evidence for nursing managers. By clarifying the pathway from perceived managerial care to feedback‐seeking behavior to reflective ability, managers can develop differentiated and context‐specific strategies, tailoring care and support measures for new nurses across departments and career stages to systematically enhance reflective ability and professional adaptability.

## 7. Limitations

Although this study provides meaningful insights into enhancing reflective ability among new nurses, several limitations should be acknowledged. First, all variables were measured using self‐report questionnaires. Although statistical methods were applied to assess CMB, such approaches cannot fully eliminate potential bias. Future studies may adopt multiple data collection methods, such as experimental designs, supervisor‐rated assessments, and qualitative interviews, to improve measurement accuracy and methodological rigor. Second, this study only examined the autoregressive and cross‐lagged relationships among three variables. Future research could incorporate additional individual and organizational factors, such as psychological safety and team climate, to provide a more comprehensive understanding of the relationships and underlying mechanisms among variables. Finally, compared with secondary and other levels of hospitals, tertiary hospitals generally possess advantages in resource allocation, training systems, and managerial support. Therefore, the applicability of the findings to other healthcare settings should be interpreted with caution. In addition, all participants in this study were from China, and cultural differences may limit the generalizability of the findings to other countries. Future studies are encouraged to expand the sampling scope by including healthcare institutions from different countries and at various levels, in order to enhance the generalizability of the results.

## 8. Conclusion

Using a longitudinal survey design and mediation analysis, this study found that perceived managerial care was associated with new nurses’ reflective ability through the mediating role of feedback‐seeking behavior. Furthermore, within the time frame of this study, perceived managerial care demonstrated a certain degree of stability during the career development of new nurses and may show a sustained positive association with both feedback‐seeking behavior and reflective ability. These findings further highlight the important role of perceived managerial care in the development of reflective ability among new nurses and reveal the potential mechanism of feedback‐seeking behavior. Based on these results, it is recommended that healthcare institutions pay greater attention to fostering supportive managerial environments. By cultivating a supportive work climate and establishing effective feedback mechanisms, organizations can encourage new nurses to engage in feedback‐seeking behavior, thereby supporting the development of their reflective ability and supporting their professional adaptation and growth.

## Author Contributions

Yongkang Fu: writing–original draft, formal analysis, and data curation.

Dongrun Liu: writing–review ad editing, conceptualization, and data curation.

Zhengyi Ma: writing–review and editing, data curation, and investigation.

Hangna Qiu: data curation, validation, and writing–review and editing.

Juntong Jing: writing–review and editing, methodology, and formal analysis.

Chaoran Chen: writing–review and editing, resources, and funding acquisition.

Enshe Jiang: writing–review and editing, supervision, and methodology.

Xiaoguang Zhang: writing–review and editing, formal analysis, and project administration.

## Funding

This work was supported by Henan Provincial Health Department Project (Grant number: LHGJ20250527) and Zhengzhou Social Science Research Project (Grant number: ZSLX20250266).

## Consent

The purpose of the study was explained to all participants before the survey was conducted, and informed consent was obtained.

## Conflicts of Interest

The authors declare no conflicts of interest.

## Data Availability

The data that support the findings of this study are available upon request from the corresponding author. The data are not publicly available due to privacy or ethical restrictions.
